# Real-world analyses of major adverse cardiovascular events and mortality risk after androgen deprivation therapy initiation in black vs. white prostate cancer patients

**DOI:** 10.1038/s41391-025-00963-y

**Published:** 2025-04-18

**Authors:** Judd W. Moul, Deborah M. Boldt-Houle, Mack Roach

**Affiliations:** 1 Department of Urology and Duke Cancer Institute, Duke University, Durham, NC USA; 2 Medical Affairs, Tolmar, Inc., Buffalo Grove, IL USA; 3 University of California San Francisco, San Francisco, CA USA

**Keywords:** Prostate cancer, Cancer epidemiology, Outcomes research

## Abstract

**Background:**

Prostate cancer(PCa) patients treated with androgen deprivation therapy(ADT) may experience major adverse cardiovascular events(MACE) [1]. Racial disparities in PCa incidence and outcomes have been noted. In contrast to older studies, three recent studies found significantly longer overall survival in Black vs. White patients: 2019 meta-analysis of nine phase III trials in men with metastatic castration-resistant PCa(CRPC) (*n* = 8820) [2]; 2020 registry study in men with metastatic CRPC (*n* = 1902) [3]; and 2023 study in men with non-metastatic CRPC (*n* = 12,992) [4]. Our “real-world” data study compared MACE and all-cause mortality risk for Black vs. White PCa patients. Compared to prior studies [1–4], our study encompassed a broader scope and was not exclusive to CRPC patients.

**Methods:**

Historical, longitudinal patient-level were collected from the Decision Resources Group (DRG, now Clarivate) Real World Evidence repository. The analysis included PCa patients receiving ≥1 ADT 1991–2020. Multivariable regression model accounted for baseline metastasis, BMI (<18.5 vs. ≥18.5 kg/m^2^), oncology vs. urology setting, antagonist vs. agonist, personal MACE history, tobacco history, baseline prostate-specific antigen (>4 vs. ≤4 ng/mL), race (White vs. Black), statin use, increasing age per year, ethnicity (non-Hispanic vs. Hispanic), increasing ADT exposure per year, diabetes, hypertension, and family MACE history.

**Results:**

MACE risk was higher for White patients than Black (4.0% vs. 2.4% at one year after ADT initiation; 21.0% vs. 13.3% at four years). Mortality risk after ADT initiation was 1.6% and 2.6% at 1 year and 11.7% and 18.1% at 4 years for Black and White patients, respectively.

**Conclusions:**

Our analysis reveals a unique finding that MACE and all-cause mortality incidence were higher in White vs. Black patients. Black race is associated with lower MACE rates and improved survival for men undergoing ADT treatment. Whether selection bias, underlying biology or other factors are responsible for these differences remains unknown.

## Introduction

Both prostate cancer (PCa) [5] and cardiovascular disease (CVD) [6] are common in the United States. Prostate cancer is the second most common cancer among men [5], and CVD is the leading cause of death [6]. Much research has been devoted to studying how prostate cancer and cardiovascular disease intertwine. Within the population of men with PCa and CVD, outcomes may vary by race. First, disparities in PCa outcomes between racial groups have been noted, with studies showing ~67% higher incidence and a greater than 2-fold increased risk of mortality in Black vs. White men [7]. Additionally, age-adjusted cardiovascular mortality rates are higher in Black vs. White men (rate ratio = 1.33, 95% CI 1.32–1.34) in the general population [8]. African American patients also demonstrate higher prostate-specific antigen (PSA) values [9] and potentially higher tumor cell burden compared to White patients at presentation.

Counter to SEER data reporting increased mortality risk in Black vs. White men with PCa [10], several studies have recently reported improved survival among Black men with PCa, albeit only in the subgroup of patients with metastatic castration-resistant PCa (mCRPC). For example, a 2023 systematic review [11] found that several studies for specific treatments (sipuleucel-T [3, 12, 13], radium [14], abiraterone alone [15], and abiraterone or enzalutamide [16, 17]) reported better survival for Black vs. White patients. As Black men have higher CVD risk than White men in the general population [8], and cardiovascular risk and mortality by race in men with PCa on androgen deprivation therapy (ADT) has not been well examined, our real-world data study explored major adverse cardiovascular events (MACE) and all-cause mortality risk for Black vs. White patients with PCa treated with ADT. Compared to prior studies [1–4], our study encompassed a broader scope and was not exclusive to men with CRPC. Based on previous data from men with mCRPC, we hypothesized that Black men may have lower MACE risk and longer/better survival after initiation of ADT, adding to the body of evidence for Black men with PCa on ADT.

## Methods

### Study design

Data were collected from the Decision Resources Group (DRG, now Clarivate) Real World Evidence repository, which links medical claims, prescription claims, and US Electronic Healthcare Records to provide historical, longitudinal patient-level data. The analysis set included PCa patients who received ≥1 ADT injection between 1991 and 2020 (99% of patients started ADT between 2010 and 2020).

### Definitions and queries

In our analysis set, only PCa patients who have taken ADT medication are considered. Prostate cancer is defined as the patient being diagnosed with PCa. The DRG extracted PCa patients from their database using keywords documented in Supplementary Table 1. Androgen deprivation therapy medication includes subcutaneous and intramuscular leuprolide, triptorelin, goserelin, histrelin, and degarelix. Similar to PCa, ADT data are extracted from the database using keywords (documented in Supplementary Table 2). Patients without any data after their earliest ADT in the DRG database are excluded.

Androgen deprivation therapy start date is the primary reference time point, and data are classified as before or after ADT start. Androgen deprivation therapy start date is defined as the date of the earliest ADT documented in the DRG database for each patient.

For MACE-related analysis, MACE is defined as the first event since ADT start. Since only the first event is considered, each patient contributes only one event maximum to the analysis i.e., MACE, which is not the first event after ADT start or happened before ADT start, is not considered in the risk analysis. Baseline values are defined as the average value within 30 days prior to ADT start, and if no value exists, the latest data point available before ADT start is used.

Major adverse CV event and all-cause mortality are the clinical outcomes we considered within our analysis. Components for MACE are extracted using keywords and ICD codes documented in Supplementary Table 3. Patients are excluded if they experienced a MACE within the 6-month (180-day) window prior to ADT start (inclusive), consistent with HERO trial methodology. Comorbidities (diabetes and hypertension) are defined as having taken medication to treat comorbidities or being diagnosed with the comorbidities prior to the first events after ADT start. Keywords and ICD codes to extract comorbidities data are documented in Supplementary Table 4. All-cause mortality is defined as a recorded “deceased” status for each patient in the database. The date of all-cause mortality is defined as the most recent date of data entry in the database for all of those who are recorded as “deceased”.

### Analysis methodology

An analysis of retrospective data from patients with PCa treated with ADT (*n* = 44,439) was performed. Kaplan–Meier curves were generated to compare risk of MACE and all-cause mortality following ADT initiation for Black and White PCa patients. MACE was defined as all-cause mortality, stroke, and myocardial infarction based on 2 recent studies in PCa patients: HERO (randomized trial comparing relugolix and leuprolide over 48 weeks) [18] and PRONOUNCE (randomized trial comparing degarelix and leuprolide in patients with PCa and concomitant athero-sclerotic cardiovascular (CV) disease over 12 months) [19]. Univariable Cox regressions to calculate the unadjusted hazard ratio (HR) and 95% confidence interval (CI) were performed by analyzing all data after first dose of ADT for each variable.

All available confounding variables were evaluated in a multivariable Cox regression analysis to calculate the adjusted HR and 95% CI. The multivariable regression model adjusted for the following variables: baseline metastasis (with vs. without), BMI (<18.5 vs. ≥18.5 kg/m^2^), treatment setting (oncology vs. urology), drug type (GnRH antagonist vs. LHRH agonist), personal MACE history, tobacco history, baseline PSA (>4 vs. ≤4 ng/mL), race (White vs. Black), statin use, increasing age per year, ethnicity (non-Hispanic vs. Hispanic), increasing ADT exposure per year, diabetes, hypertension, and family MACE history. Another multivariable regression model adjusted for 2 variables: BMI (<18.5 vs. ≥18.5 kg/m^2^) and race (White vs. Black) to identify whether and how much the 2 variables accounted for the other factor’s risk of MACE and/or mortality.

Body mass index was analyzed using two approaches: as a continuous variable and as a categorical variable. While both methods were employed, the group with a BMI < 18.5 kg/m^2^ demonstrated the largest absolute difference relative to other BMI groups. This difference was also statistically significant compared to all other BMI groups in the univariable analysis, and therefore BMI < 18.5 kg/m^2^ vs ≥18.5 kg/m^2^ was selected as the categorical variable for univariable and multivariable analysis.

## Results

### Demographics

**Fig. 1:**
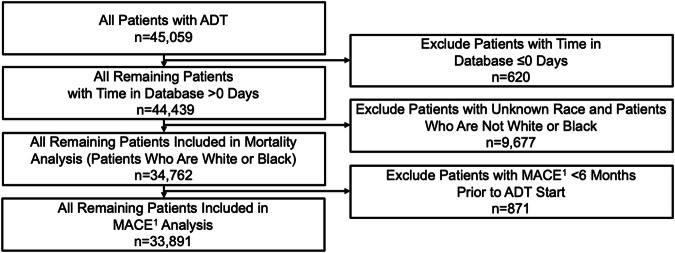
Consort diagram. ^1^MACE (recent urology studies HERO and PRONOUNCE definition) defined as myocardial infarction, stroke, and mortality from any cause. 34,762 and 33,891 patients were included in the mortality and MACE analyses, respectively. MACE Major adverse cardiovascular events, ADT Androgen deprivation therapy, MI Myocardial infarction.

34,762 patients were included in the Black vs. White analyses (Fig. 1) and 5817 were Black (Table 1). The proportion of Black patients included in our analysis (13%) is representative of the US population (14%) [20].

**Table 1:** Baseline demographics of black vs white patients with prostate cancer.

Categories		Total White/Black *N* = 34,762		White *N* = 28,945		Black *N* = 5817	
Age	Mean (SD)	73.8	(8.2)	74.3	(8.0)	71.2	(8.6)
	Median (25–75%)	75.0	(68–81)	75.0	(69–81)	71.0	(65–78)
Ethnicity	Hispanic, %	2.4		2.8		0.6	
	Non-Hispanic, %	86.3		86.4		85.7	
	Unknown,%	11.3		10.9		13.6	
Race	White, %	83.3		100.0		0.0	
	Black, %	16.7		0.0		100.0	
	Asian, %	0.0		0.0		0.0	
	Other, %	0.0		0.0		0.0	
	Unknown,%	0.0		0.0		0.0	
Metastasis	Baseline, %	2.5		2.7		1.6	
Baseline body mass index (kg/m^**2**^)	≥35, %	10.2		10.1		10.8	
	30–<35, %	19.4		19.6		18.4	
	25–<30, %	33.6		34.5		28.9	
	18.5–<25, %	17.4		17.3		17.7	
	<18.5, %	0.7		0.5		1.4	
	Unknown, %	18.8		18.0		22.8	
	Mean (SD)	28.8	(4.9)	28.8	(4.9)	28.8	(5.4)
	Median (25–75%)	28.2	(25.3–31.8)	28.2	(25.4–31.8)	28.3	(25.0–32.2)
Baseline prostate-specific antigen (ng/mL)	>4, %	26.6		25.4		32.5	
	≤4, %	23.9		24.4		21.2	
	Unknown, %	49.5		50.2		46.2	
	Mean (SD)	39.5	(286.8)	34.7	(267.2)	61.7	(363.2)
	Median (25–75%)	4.6	(0.2–13.9)	4.2	(0.1–12.8)	6.5	(0.6–19.8)
Comorbidity	Diabetes, %	19.6		18.9		23.4	
	Hypertension, %	79.6		79.4		80.3	
	Hypercholesterolemia, %	63.3		64.8		56.2	
Statin use	Yes, %	52.9		53.9		47.8	
Personal history	MACE, %	5.6		5.9		4.5	
Family history	MACE, %	6.6		7.1		4.0	
Tobacco use history	Yes, %	9.6		9.2		11.3	
	No, %	57.1		58.2		51.7	
	Unknown, %	33.4		32.6		37.0	
Urology/Oncology	Urology, %	49.7		48.7		54.8	
	Oncology, %	3.9		4.1		3.2	
	Both, %	14.6		13.5		20.0	
	Unknown, %	31.8		33.7		21.9	

*CV* Cardiovascular, *ADT* Androgen deprivation therapy.

34,762 patients were included in the Black vs. White analyses and 5817 were Black. The proportion of Black patients included in our analysis (13%) is representative of the US population (14%).

### MACE

**Fig. 2:**
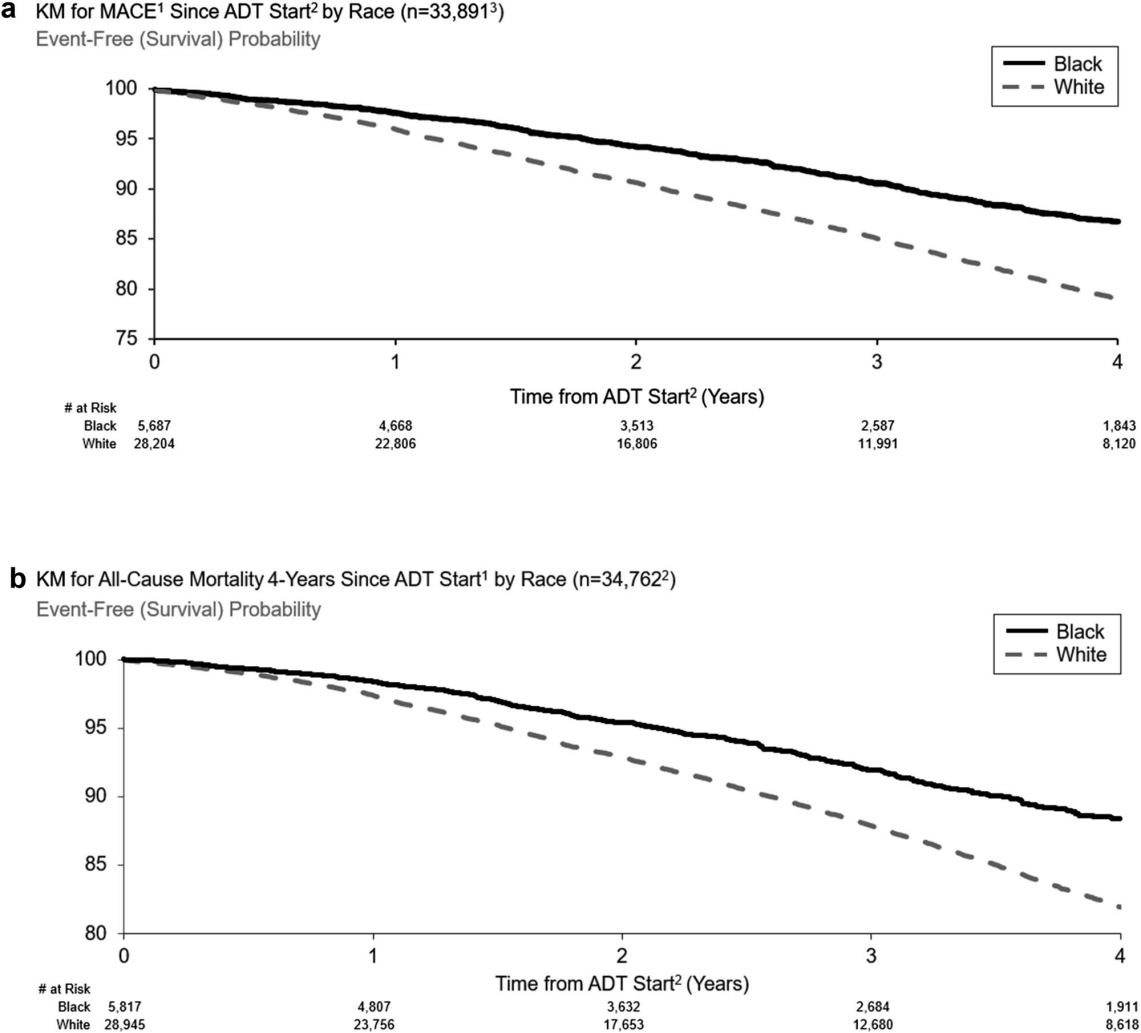
Kaplan-Meier Curves for MACE and All-Cause Mortality 4-Years Since ADT Start by Race. **a** MACE risk was higher for White patients than Black (4.0% and 2.4%, for White and Black patients, respectively at one year after ADT initiation; 21.0% and 13.3%, respectively, at four years). MACE Major adverse cardiovascular events, ADT Androgen deprivation therapy, LHRH Luteinizing hormone-releasing hormone. **b** The mortality risk after ADT initiation was 1.6% and 2.6% at 1 year and 11.7% and 18.1% at 4 years for Black and White patients, respectively. ADT Androgen deprivation therapy, LHRH Luteinizing hormone-releasing hormone. **a** Kaplan–Meier Curve for MACE Since ADT Start by Race. 1 Major Adverse Cardiovascular Events (recent urology studies HERO and PRONOUNCE definition) defined as MI, stroke, and mortality from any cause. 2 Date of earliest LHRH injection recorded for patient 3 Excluded patients who had a MACE < 6 months prior to ADT Start. **b** Kaplan–Meier Curve for All-Cause Mortality 4-Years Since ADT Start by Race. 1 Date of earliest LHRH injection recorded for patient. 2 Excluded patients with no race data.

MACE risk was higher for White patients than Black (4.0% and 2.4%, for White and Black patients, respectively at 1 year after ADT initiation; 21.0% and 13.3%, respectively, at 4 years) (Fig. 2a). The unadjusted and adjusted HRs for MACE risk in White vs. Black patients were 1.68 (95% CI 1.56–1.82, *p* < 0.001) and 1.30 (95% CI 1.09–1.56, *p* < 0.05), respectively.

When evaluating only the impact of BMI and race on MACE, unadjusted MACE risk was higher for patients with BMI < 18.5 vs. ≥18.5 kg/m^2^ (HR = 2.29, 95% CI 1.82–2.90, *p* < 0.001) and for White vs. Black patients (HR = 1.69, 95% CI 1.56–1.82, *p* < 0.001) (Table 2). In the bivariate analysis evaluating BMI and race, adjusted MACE risk was higher for patients with BMI < 18.5 vs. BMI ≥ 18.5 kg/m^2^ (HR = 2.45, 95% CI 1.87–3.22, *p* < 0.001) and for White vs. Black patients (HR = 1.72, 95% CI 1.56–1.89, *p* < 0.001).

**Table 2:** Hazard ratio (95% CI) and *P*-value of MACE for factors in univariate and multivariable analysis.

Factors	Univariable			Multivariable (*N* = 27,398^b,c,d^)	
	*n*	HR (95% CI)	*P*-value	HR (95% CI)	*P*-value
BMI < 18.5 vs ≥18.5 (kg/m^2^)	34,861^b,c^	2.29 (1.82–2.90)	<0.001	2.45 (1.87–3.22)	<0.001
White vs Black (Race)	33,891^b,d^	1.69 (1.56–1.82)	<0.001	1.72 (1.56–1.89)	<0.001

MACE risk was higher for White vs. Black patients and patients with BMI <18.5 vs. BMI ≥18.5 kg/m^2^. BMI and race are largely independent and do not account for the other factor’s increased MACE risk.

*CI* Confidence interval, *MACE* Major adverse cardiovascular events, *HR* Hazard ratio, *BMI* Body mass index.

^a^MACE (recent urology studies HERO and PRONOUNCE definition) defined as myocardial infarction, stroke, and mortality from any cause.

^b^Excluded patients who had a MACE <6 months prior to ADT Start.

^c^Excluded patients without BMI data.

^d^Excluded patients who were not of white or black race.

### Mortality

Mortality risk was higher for White patients than Black (2.6% and 1.6% for White and Black patients, respectively, at 1 year after ADT initiation; 18.1% and 11.7%, respectively, at 4 years) (Fig. 2b). The unadjusted and adjusted HRs for all-cause mortality risk in White vs. Black patients were 1.66 (95% CI 1.53–1.80, *p* < 0.001) and 1.24 (95% CI 1.01–1.52, *p* < 0.05), respectively.

When evaluating only the impact of BMI and race on all-cause mortality, unadjusted mortality risk was higher for patients with BMI < 18.5 vs. ≥18.5 kg/m^2^ (HR = 2.56, 95% CI 2.03–3.24, *p* < 0.001) and for White vs. Black patients (HR = 1.66, 95% CI 1.53–1.80, *p* < 0.001) (Table 3). In the bivariate analysis evaluating BMI and race, adjusted mortality risk was higher for patients with BMI < 18.5 vs. BMI ≥ 18.5 kg/m^2^ (HR = 2.91, 95% CI 2.23–3.80, *p* < 0.001) and White vs. Black patients (HR = 1.67, 95% CI 1.51–1.84, *p* < 0.001).

**Table 3:** Hazard ratio (95% CI) and *P*-value of all-cause mortality for factors in univariate and multivariable analysis.

Factors	Univariable			Multivariable (*N* = 28,231^a,b^)	
	*n*	HR (95% CI)	*P*-value	HR (95% CI)	*P*-value
BMI < 18.5 vs ≥18.5 (kg/m^2^)	35,918^a^	2.56 (2.03–3.24)	<0.001	2.91 (2.23–3.80)	<0.001
White vs Black (Race)	34,762^b^	1.66 (1.53–1.80)	<0.001	1.67 (1.51–1.84)	<0.001

Mortality risk was higher for White vs. Black patients and patients with BMI <18.5 vs. BMI ≥18.5 kg/m^2^. BMI and race are largely independent and do not account for the other factor’s increased mortality risk.

*CI* Confidence interval, *HR* Hazard ratio, *BMI* Body mass index.

^a^Excluded patients without BMI.

^b^Excluded patients who were not of white or black race.

## Discussion

Previously published studies have evaluated outcomes by race in patients with PCa [2–4, 21–30]. A 2023 review of PCa, race, and healthy disparity [31] included nine studies that demonstrated a racial disparity in the screening, early detection, and treatment of PCa [32–40], with findings such as Black men are less likely to receive aggressive PCa treatment [32] and definitive treatment by radiation or surgery [33, 39], and Black men have less knowledge of PCa and early detection [35]. This review also reported that, of 12 phase 3 randomized clinical trials reporting outcomes by race, only one suggested a worse outcome for African American men [24]. Additionally, three studies support longer/better overall survival in Black vs. White patients with CRPC: a 2019 meta-analysis of nine phase III trials in men with metastatic CRPC (*n* = 8820) [2]; a 2020 registry study in men with metastatic CRPC (*n* = 1902) [3]; and a 2023 study in patients with non-metastatic CRPC (*n* = 12,992) [4]. Another study analyzing data from five phase III randomized radiotherapy PCa trials (*n* = 5624) found similar 10-year overall survival rates in Black vs. White patients (58% vs. 60%) [41]. Expanding on these previously published studies [2–4], our real-world data study evaluated the two separate endpoints of all-cause mortality risk by race and MACE risk by race in patients with PCa on ADT.

Compared to prior studies, our study encompassed a broader scope and was not exclusive to men with CRPC. Our analysis of data over the most recent decade from ~45,000 PCa patients is likely an accurate reflection of the real world.

Contrary to expectations based on SEER data reporting increased mortality risk in Black vs. White patients with PCa [10], our analysis found that both MACE and all-cause mortality were lower in Black vs. White patients with PCa on ADT. This is consistent with other literature suggesting improved outcomes among Black patients with CRPC compared to non-Hispanic White patients [15, 42]. Adding to this body of evidence for Black men with PCa, our research found that Black race is associated with lower MACE rates and improved overall survival for men undergoing treatment with ADT. One potential explanation for our findings is that White men had higher baseline rates of MACE compared to Black men, which could have led to increased MACE risk. Another potential hypothesis for higher mortality risk in White patients is survival bias of Black PCa patients. As Black adults have a significantly younger age of cardiovascular disease diagnosis compared to White adults (50 vs. 56 years) [43], it is possible that Black patients with higher MACE risk passed away before developing PCa and were not captured in the database used for this study. A third potential hypothesis for higher mortality in White vs. Black patients is that Black patients have denser lean body mass than White patients [44], which can protect them from cancer cachexia and allow for longer/better overall survival. Indeed, consistent with previous literature reporting better prognosis in patients with cardiovascular disease who were classified as overweight or obese [45], our analysis results suggest that BMI contributes to improved survival (and lower MACE) independent of race. Of course, the categorical variable of BMI < 18.5 kg/m^2^ is “underweight” [46] and may be proxy for cancer cachexia rather than a more direct lean/obesity related mechanism. Finally, a fourth potential hypothesis for higher mortality in White vs. Black patients is the *HSD3B1* (1245C) allele inheritance, which can cause ADT resistance in men with PCa [47] and, thereby, lower overall survival [47, 48]. This germline is more prevalent in White (10%) than Black (1%) populations [48]. Therefore, White race could be considered a predisposing risk factor for MACE and mortality in PCa patients undergoing ADT.

A multi-disciplinary care team (e.g., primary care physician, urologist, oncologist, cardiologist/cardio-oncologist) should collaborate in patient care, with the goal of providing optimal CV treatment in all PCa patients. Additionally, physicians should actively manage lifestyle habits, including dietary habits, to further mitigate CV risks and improve patient outcomes. Indeed, within the context of phase 3 trials [19, 49], providing optimal cardiac care can minimize the MACE inducing effects of ADT. As such, ensuring all patients have equal access will go a long way to improving outcomes among all patients, especially those with decreased access to care (i.e., Black patients).

Our study has limitations. First, retrospective database studies are hypothesis-generating rather than confirmatory. However, the large size (~45,000 patients from a database containing >300 million patients), long follow-up (10 years for some patients), recent clinical experience (99% from 2010–2020), and diversity of the dataset give weight to the results being an accurate representation of current clinical experience.

Second, our findings are based on a database that used ICD codes, thus the reliability is limited by the accuracy of coding practices. However, any misclassification bias would likely occur uniformly across treatment groups. Third, several other PCa treatments, such as ARI and the co-administration of glucocorticoid [50], have been found to be associated with cardiovascular risk in patients. This may have been a confounding factor and future studies are needed. Additionally, the MACE and mortality analyses are not truly independent since mortality is part of MACE and takes up a large proportion of MACE.

Lastly, socioeconomic factors were not assessed and may be potential confounders. Socioeconomic factors impact patients’ ability to afford and access healthcare, and it has been established that low socioeconomic status is associated with higher risk of morbidity and mortality due to CV disease [51]. It should also be noted that “race” is a social construct. Thus, any differences by “race” are likely due to social factors e.g., systemic racism, lower socioeconomic status, food deserts, lack of health insurance, and lack of preventative care. However, given the real-world knowledge of the distribution of these factors, we might expect Black men to experience higher MACE and mortality risks. That said, equal access to care would likely attenuate differences in survival by “race” and is an important determinant of racial equity. Indeed, a study of over 60,000 men with PCa treated in the equal-access Veterans Affairs medical system found that the 10-year PCa-specific mortality rate was slightly lower for African American men compared to Non-Hispanic White men (4.4 vs. 5.1%; *p* = 0.005) [52]. These data, along with our study, suggest that, in contrast to national trends, African American men diagnosed with PCa do not appear to present with more advanced disease or experience worse outcomes in comparison to Non-Hispanic White Men. Future studies should analyze factors such as social determinants of health, regional differences, types of insurance, and annual income, to reduce potential bias to due socioeconomic factors.

## Conclusion

Our analysis reveals a unique finding that both MACE and all-cause mortality incidence were higher in White vs. Black patients. BMI and race are largely independent and do not account for the other factor’s increased MACE and mortality risk. Adding to the body of evidence for Black men with PCa, our research reveals that the Black race is associated with lower MACE rates and improved survival in men on ADT. Potential explanations for these findings may include genetic factors or other hypotheses warranting further investigation. Future studies should evaluate the role of co-morbidities on MACE risk for PCa patients during ADT to identify other CV predictors, confirm our findings that White PCa patients have higher MACE and mortality risk compared to Black patients, and investigate the above hypotheses. Finally, despite the common belief dating back more than 20 years, Black men with PCa do not appear to have inherently higher risks of MACE and mortality.

## Supplementary information

Supplemental Table 1

Supplemental Table 2

Supplemental Table 3

Supplemental Table 4

## Data Availability

The data that support the findings of this study are available from the corresponding author upon reasonable request. Restrictions apply to the availability of data generated or analyzed during this study to preserve patient confidentiality or because they were used under license. The corresponding author [judd.moul@duke.edu] will on request detail the restrictions and any conditions under which access to some data may be provided.
