# Supplementary material for: Real-world analyses of major adverse cardiovascular events and mortality risk after androgen deprivation therapy initiation in black vs. white prostate cancer patients
Source: Prostate Cancer Prostatic Dis. 2025 Apr 18;28(4):946–52. doi: 10.1038/s41391-025-00963-y (PMC12643916; doi:10.1038/s41391-025-00963-y)
Supplement: Supplementary file 1 — Supplemental Table 1 [file 41391_2025_963_MOESM1_ESM.docx]

Supplementary Table 1: Prostate Cancer Inclusion Criteria by DRG

| **Prostate Cancer Type** | **ICD-9** | **ICD-10** |
| --- | --- | --- |
| Malignant neoplasm of prostate | 185 | C61 |
| Secondary malignant neoplasm of genital organs | 198.82 | C79.82 |
| Carcinoma *in situ* of prostate | 233.4 | D07.5 |
