# Supplementary material for: Real-world analyses of major adverse cardiovascular events and mortality risk after androgen deprivation therapy initiation in black vs. white prostate cancer patients
Source: Prostate Cancer Prostatic Dis. 2025 Apr 18;28(4):946–52. doi: 10.1038/s41391-025-00963-y (PMC12643916; doi:10.1038/s41391-025-00963-y)
Supplement: Supplementary file 2 — Supplemental Table 2 [file 41391_2025_963_MOESM2_ESM.docx]

Supplementary Table 2 – Keywords to Extract ADT Data

| **Generic Name(s)** | **Brand Name(s)** |
| --- | --- |
| Leuprolide | ELIGARD, LUPRON |
| Goserelin | ZOLADEX |
| Triptorelin | TRELSTAR |
| Degarelix | FIRMAGON |
| Histrelin | VANTAS, SUPPRELIN |
