# Supplementary material for: Real-world analyses of major adverse cardiovascular events and mortality risk after androgen deprivation therapy initiation in black vs. white prostate cancer patients
Source: Prostate Cancer Prostatic Dis. 2025 Apr 18;28(4):946–52. doi: 10.1038/s41391-025-00963-y (PMC12643916; doi:10.1038/s41391-025-00963-y)
Supplement: Supplementary file 3 — Supplemental Table 3 [file 41391_2025_963_MOESM3_ESM.docx]

Supplementary Table 3.1: Keywords Used to Extract CV Events

| **Keywords** |
| --- |
| AMI, Arrhythmia, Atrial, Attack, Cardiac, Cardiovascular, Cerebral, CV (CVD), Fibrillation, Heart, HF, Infarction, Ischemic, MACE, MI, Myocardial, Stroke, Tachycardia, Vascular, Ventricular |

Supplementary Table 3.2: ICD Codes Used to Extract CV Events

| **CV Event** | **ICD-9** | **ICD-10** |
| --- | --- | --- |
| Myocardial Infarction | 410, 412 | I21, I22, I23, I25.2 |
| Stroke | 423, 430, 431, 432, 433, 434, 435, 436, 437 | G45, I64, I65, I66 |
| Unstable Angina | NA | I200 |
| Arterial Disease/Peripheral Vascular Disease | 17.56, 39.25, 39.26, 39.29, 39.5, 39.9, 411, 413, 414 | I24.0 |
| Heart Failure | NA | I09.9, I11, I13.0, I13.2, I25.5, I42.0, I42.5, I42.6, I42.7, I42.8, I42.9, I43, I50, I97.1, K76.1, P29.0 |
