# Supplementary material for: Real-world analyses of major adverse cardiovascular events and mortality risk after androgen deprivation therapy initiation in black vs. white prostate cancer patients
Source: Prostate Cancer Prostatic Dis. 2025 Apr 18;28(4):946–52. doi: 10.1038/s41391-025-00963-y (PMC12643916; doi:10.1038/s41391-025-00963-y)
Supplement: Supplementary file 4 — Supplemental Table 4 [file 41391_2025_963_MOESM4_ESM.docx]

Supplementary Table 4.1: Comorbidity Inclusion Criteria – Diabetes

| **Code Description** | **Code(s) or Keyword(s)** |
| --- | --- |
| Diagnosis Keywords | Diabetes, Diabetes Insipidus, Type 1 Diabetes, Type 2 Diabetes |
| Medication Keywords | Acarbose, ActoPlus Met, Actos, Adlyxin, Admelog, Afrezza, Albiglutide, Alogliptin, Amaryl, Apidra, Avandamet, Avandaryl, Avandia, Basaglar, Bromocriptine, Bydureon, Bydureon Bcise, Byetta, Canagliflozin, Chlorpropamide, Colesevelam, Cycloset, Dapagliflozin, Diabeta, Diabinese, Duetact, Dulaglutide, Empagliflozin, Ertugliflozin, Exenatide, Farxiga, Fiasp, Fortamet, Glimepiride, Glipizide, Glipizide XL, Glucophage, Glucophage XR, Glucotrol, Glucotrol XL, Glucovance, Glumetza, Glyburide, Glycron, Glynase, Glynase Prestab, Glyset, Glyxambi, Humalog, Humalog Kwikpen, Humulin, Humulin 70 / 30, Humulin n, Humulin r, Humulin r u-500, Humulin r u-500 kwikpen, Insulin Aspart, Insulin Degludec, Insulin Detemir, Insulin Glargine, Insulin Glulisine, Insulin Isophane, Insulin Lispro, Insulin Regular, Invokamet, Invokamet XR, Invokana, Janumet, Janumet XR, Januvia, Jardiance, Jentadueto, Kazano, Kombiglyze, Kombiglyze XR, Kwikpen, Lantus, Lantus Solostar, Levemir, Linagliptin, Liraglutide, Lixisenatide, Lyumjev, Metaglip, Metformin, Miglitol, Myxredlin, Nateglinide, Nesina, Novolin, Novolin n, Novolin r, Novolog, Novolog Flexpen, Novolog Penfill, Omeprazole, Onglyza, Oseni, Ozempic, Pioglitazone, Pramlintide, pramlintide acetate, PrandiMet, Prandin, Precose, Qtern, Repaglinide, Riomet, Rosiglitazone, Rxnx, Rybelsus, Saxagliptin, Segluromet, Semaglutide, Semglee, Sitagliptin, Soliqua, Starlix, Steglatro, Steglujan, Symlin, SymlinPen, SymlinPen 120, SymlinPen 60, Tanzeum, Tolazamide, Tolbutamide, Toujeo Max Solostar, Toujeo Solostar, Tradjenta, Tresiba, Trulicity, Victoza, Welchol, Xigduo XR |
| ICD-9 | 249, 250 |
| ICD-10 | E08, E09, E10, E11, E12, E13 |

Supplementary Table 4.2: Comorbidity Inclusion Criteria – Hypertension

| **Code Description** | **Code(s) or Keyword(s)** |
| --- | --- |
| Diagnosis Keywords | Hypertension, HTN, Hypertensive, High Blood Pressure |
| Medication Keywords | Aartia, Accupril, Accuretic, Acebutolol, Aceon, Adalat CC, Afeditab, Aldactazide, Aldactone, Aldoril, Aliskiren, Altace, Amiloride, Amlobenz, Amlodipine, Apresoline, Aquazide, Atacand, Atenolol, Atorvastatin, Avalide, Avapro, Azilsartan, Azor, Benazepril, Bendroflumethiazide, Benicar, Betaxolol, Bisoprolol, Brevibloc, Bystolic, Caduet, Calan, Candesartan, Capoten, Captopril, Cardene, Cardizem, Cardura, Carospir, Cartia, Carvedilol, Catapres, Chlorothiazide, Chlorthalidone, Clevidipine, Cleviprex, Clonidine, Conjupri, Consensi, Coreg, Corgard, Corzide, Covera HS, Cozaar, Demadex, Dibenzyline, Dilacor XR, Dilt, Diltia, Diltiazem, Diltzac, Diovan, Diovan HCT, Diuril, Diuril Sodium, Doxazosin, Dutoprol, Dyazide, DynaCirc CR, Edarbi, Edarbyclor, Enalapril, Enalaprilat, Enduron, Epaned, Eplerenone, Eprosartan, Esidrix, Esmolol, Exforge, Exforge HCT, Felodipine, Fosinopril, Furosemide, Guanabenz, Guanfacine, Hydralazine, Hydrochlorothiazide, Hydroflumethiazide, Hytrin, Hyzaar, Indapamide, Inderal, Inderal LA, Innopran, Inspra, Irbesartan, Isoptin SR, Isradipine, Kapspargo Sprinkle, Katerzia, Labetalol, Lasix, Levamlodipine, Levatol, Lisinopril, Lopressor, Losartan, Lotensin, Lotrel, Maleate, Matzim, Mavik, Maxzide, Mecamylamine, Methyclothiazide, Methyldopa, Metolazone, Metoprolol, Micardis, Microzide, Midamor, Minipress, Minitran, Minoxidil, Moexipril, Monopril, Nadolol, Naturetin, Nebivolol, Nicardipine, Nifedipine, Nimodipine, Nisoldipine, Nitro TD Patch-A, Nitro-Bid, Nitro-Dur, Nitro-Time, Nitroglycerin, Nitrolingual Pumpspray, Nitromist, Nitrostat, Norvasc, Olmesartan, Oretic, Penbutolol, Perindopril, Phenoxybenzamine, Pindolol, Plendil, Prazosin, Prestalia, Prinivil, Procardia, Propranolol, Qbrelis, Quinapril, Ramipril, Saluron, Sectral, Spironolactone, Sular, Tarka, Tartrate, Taztia, Tekturna, Telmisartan, Telmisartan Eprosartan, Tenex, Tenoretic, Tenormin, Terazosin, Teveten, Thalitone, Tiadylt, Tiazac, Timolol, Toprol, Toprol XL, Torsemide, Trandate, Trandolapril, Tribenzor, Twynsta, Univasc, Valsartan, Vaseretic, Vasotec, Vecamyl, Verapamil, Verelan, Zaroxolyn, Zebeta, Zestoretic, Zestril, Ziac |
| ICD-9 | 401, 402, 403, 404, 405 |
| ICD-10 | I10, I11, I12, I13, I14, I15 |
